# Theoretical Prediction and Experimental Verification of Protein-Coding Genes in Plant Pathogen Genome *Agrobacterium tumefaciens* Strain C58

**DOI:** 10.1371/journal.pone.0043176

**Published:** 2012-09-11

**Authors:** Qian Wang, Yang Lei, Xiwen Xu, Gejiao Wang, Ling-Ling Chen

**Affiliations:** 1 State Key Laboratory of Agricultural Microbiology, College of Life Science and Technology, Huazhong Agricultural University, Wuhan, People's Republic of China; 2 Center for Bioinformatics, Huazhong Agricultural University, Wuhan, People's Republic of China; Virginia Tech, United States of America

## Abstract

*Agrobacterium tumefaciens* strain C58 is a Gram-negative soil bacterium capable of inducing tumors (crown galls) on many dicotyledonous plants. The genome of *A. tumefaciens* strain C58 was re-annotated based on the Z-curve method. First, all the ‘hypothetical genes’ were re-identified, and 29 originally annotated ‘hypothetical genes’ were recognized to be non-coding open reading frames (ORFs). Theoretical evidence obtained from principal component analysis, clusters of orthologous groups of proteins occupation, and average length distribution showed that these non-coding ORFs were highly unlikely to encode proteins. Results from the reverse transcription-polymerase chain reaction (RT-PCR) experiments on three different growth stages of *A. tumefaciens* C58 confirmed that 23 (79%) of the identified non-coding ORFs have no transcripts in these growth stages. In addition, using theoretical prediction, 19 potential protein-coding genes were predicted to be new protein-coding genes. Fifteen (79%) of these genes were verified with RT-PCR experiments. The RT-PCR experimental results confirmed the reliability of our theoretical prediction, indicating that false-positive prediction and missing genes always exist in the annotation of *A. tumefaciens* C58 genome. The improved annotation will serve as a valuable resource for the research of the lifestyle, metabolism, and pathogenicity of *A. tumefaciens* C58. The re-annotation of *A. tumefaciens* C58 can be obtained from http://211.69.128.148/Atum/.

## Introduction

*Agrobacterium tumefaciens* is a Gram-negative bacteria belonging to the *Rhizobiaceae* family. As ubiquitous soil microorganisms, most of the *A. tumefaciens* members are ideal vectors for plant gene-transfer. The products of a series of virulence (*vir*) genes export the single-stranded transferred DNA (T-DNA) in tumor-inducing (Ti) plasmid to plant cells, and the T-DNA can integrate into the plant genome randomly [1], [2]. Moreover, most of the gene sequences can be replaced by the T-DNA, making *A. tumefaciens* an essential tool for plant transgenic research. The genome of *A. tumefaciens* C58 was sequenced in 2001 by Washington University and Cereon genomics company [3], [4]. As a powerful transgenic tool, the detailed genomic study of *A. tumefaciens* C58 could lead to a directed refinement of plant transformation. The genome of *A. tumefaciens* C58 is approximately 5.67 Mb and is composed of four replicons, i.e., one circular chromosome, one linear chromosome, and two plasmids, namely, pTiC58 and pAtC58. GenBank accession numbers for the four replicons are AE007869 to AE007872. Shortly after the publication of *A. tumefaciens* C58 genome, the Comprehensive Microbial Resource of The Institute for Genomic Research automatically re-annotated it (http://cmr.jcvi.org/) and identified additional >1,000 suspicious protein-coding genes. The Reference Sequence (RefSeq) collection in the National Center for Biotechnology Information (NCBI) aims to provide a comprehensive, integrated, non-redundant, and well-annotated set of sequences, including genomic DNA, transcripts, and proteins [5]. The four *A. tumefaciens* C58 replicons were processed by RefSeq pipeline and assigned with a project number (ID: 57865). The annotation of *A. tumefaciens* C58 in the aforementioned public databases is quite different, indicating that its genome annotation is far from satisfactory.

Considering that most of the protein-coding genes annotated with gene-finding programs have not been verified experimentally, annotations in the sequenced genomes always contain false-positive and false-negative prediction, especially in the GC-rich genomes [6]–[15]. False-positive prediction indicates that some open-reading frames (ORFs) are incorrectly predicted to be protein-coding genes (most of them are short ORFs with no functional information), whereas false-negative annotation indicates true protein-coding genes missed in the genome annotation. Current gene-finding programs perform relatively well in low GC content genomes, but the accuracy drops considerably in high GC content genomes because they contain fewer overall stop codons and more spurious ORFs. False-positive prediction is a very serious problem in high GC content genomes. Given that *A. tumefaciens* C58 has a relatively high overall GC content (59.1%), this species may contain false-positive and false-negative ORFs. Klüsener *et al.* performed proteomic and transcriptomic analyses of phosphatidylcholine (PC)-deficient and wild-type *A. tumefaciens* and observed that the loss of PC can alter the expression of approximately 13% of the genes [16]. Other proteomic studies predicted that approximately 3,000 cytosolic proteins and 400 membrane proteins can be expressed under the conditions of isoelectric point (pI) 4 to 7 and a molecular weight of 10 kDa to 150 kDa. However, the proteomic experimental results detected only approximately 1,500 proteins under the above conditions [17] [18].

In the current analysis, all the *A. tumefaciens* C58 ‘hypothetical genes’ in RefSeq annotation were re-identified, 29 of these molecules were recognized as non-coding ORFs by an algorithm based on the Z-curve method [19] [20]. Evidence obtained from the principal component analysis (PCA), clusters of orthologous groups of proteins (COG) occupation, and average length distribution showed that the identified non-coding ORFs were highly unlikely to encode proteins. Reverse transcription-polymerase chain reaction (RT-PCR) experiments confirmed that 23 (79%) ORFs did not express in three important bacterial growth stages. In addition, 19 potential new protein-coding genes were predicted by two *ab initio* gene finding program and our algorithm. All the potential new protein-coding genes were tested using RT-PCR, and 15 (79%) of these genes were confirmed. Although missing genes is not the most serious problem in bacterial gene annotation, the current analysis confirmed that some protein-coding genes are still missed in the annotation. The improved annotation provides valuable information for the genomic analysis of *A. tumefaciens* C58.

## Materials and Methods

### Data collection

The sequence and annotation of *A. tumefaciens* C58 genome were downloaded from the NCBI RefSeq because it can provide a comprehensive and relatively precise annotation [5]. The ∼5.67 Mb genome of *A. tumefaciens* C58 contained four replicons, namely, a circular (2,841,580 bp, NC_003062) and a linear (2,075,577 bp, NC_003063) chromosomes as well as two plasmids, pAtC58 (542,868 bp, NC_003064) and pTiC58 (214,233 bp, NC_003065). The four replicons annotated 2765, 1851, 542, and 197 protein-coding genes, respectively.

The annotation of protein-coding genes can be classified into two groups. The first group contains genes with confirmed functions, which are used for the training dataset. The second group includes ‘hypothetical genes’ whose coding status was not determined, but are re-identified in the current analysis. A total of 2,987 function confirmed genes in the two chromosomes were used for the training dataset. The coding status of 1,071, 558, 214, and 59 ‘hypothetical ORFs’ in the circular chromosome, linear chromosome, pAtC58, and pTiC58 were re-annotated, respectively. Furthermore, potential new protein-coding genes that were not found in the RefSeq annotation were predicted by two *ab initio* gene finding programs, i.e., Prokaryotic dynamic programming gene-finding algorithm (Prodigal) [21] and FgenesB (http://linux1.softberry.com/berry.phtml?topic=fgenesb&group=programs&subgroup=gfindb), respectively.

### Identification of non-coding ORFs from annotated hypothetical genes

The method adopted in this study is based on the Z-curve of the DNA sequence, which has been applied successfully to find genes in bacterial and archaeal genomes [19] [20]. In the present analysis, 21 variables are adopted, which include 9 phase-dependent single nucleotides and 12 phase-independent di-nucleotides. Details about these variables and the identification process are listed in the Methods S1.

### Method for identifying new functional genes

In the current analysis, two *ab initio* gene finding programs are performed to identify new protein-coding genes not found in the RefSeq annotation. Prodigal is a recently developed highly accurate microbial gene finding program, with high speed, low false positive rate, and high accuracy in locating the translation initiation sites (TISs) [21]. FgenesB is another accurate *ab initio* prokaryotic gene prediction program based on the Markov chain models of coding regions, translation, and termination sites. This program also includes a simplified prediction of operons based only on distances between predicted genes. Combining the predicted result of the two *ab initio* programs and their Z scores in the Z-curve method, new protein-coding genes not annotated in RefSeq are identified in *A. tumefaciens* C58 genome.

### Strain cultivation and nucleic acid isolation

*A. tumefaciens* C58 was inoculated into 100 mL Luria-Bertani (peptone, 10 g/L; yeast extract, 5 g/L; NaCl, 10 g/L) broth and incubated at 28°C overnight with 180 rpm shaking. The bacterial cells were sampled in early log, late log, and stationary stages with OD_600_ of 0.3, 0.6, and 0.9, respectively. Total DNA was purified by phenol-chloroform-isoamyl alcohol (25∶24∶1, v/v/v) and precipitated by ethanol in the late log stage of bacterial cells. After being washed twice using 70% ethanol, the total DNA was dissolved in 100 µL sterilized H_2_O [22]. For the three growth stages, total RNA was extracted used Trizol (Invitrogen) and treated with DNase I (Takara) to remove genomic DNA contamination. Then, cDNA was made by reverse transcription using the total RNA with First Strand cDNA Synthesis Kit (Fermentas). The total DNA, total RNA, and cDNA were used for PCR analysis.

### RT-PCR and sequence validation

The 50 µL PCR mixture contained 5 µL 10×PCR Buffer, 0.2 mM dNTP (Takara), 0.02 µM primers, 1 µL total DNA, total RNA or cDNA, 1 µL taq DNA polymerase (Takara), and nuclease-free water. The samples were incubated with the following cycles: 95°C for 5 min, 30 cycles of 95°C for 45 s, annealing for 45 s, 72°C for 1 min, and a final extension of 72°C for 10 min. The PCR primers for the chosen sequences were designed by Primer Premier 5.0 software (Premier Biosoft International, Palo Alto, CA). The PCR reaction condition for every primer sit was optimized with DNA sample in repeated PCR experiments at 2°C to 10°C lower than the predicted annealing temperature until a single amplified band was obtained. The 16S rRNA gene and *recA* gene encoding recombination protein A were used as positive controls for multi-copy and single-copy genes from *A. tumefaciens* strain C58, respectively. Meanwhile, translation initiation factor gene IF-2 (named PC-3) was used as a known positive control gene in the verification experiments of protein-coding genes.

Each of the PCR products was purified using a PCR product purification kits (SBS Genetech CO., Ltd. Shanghai, China). The purified DNA fragments were ligated with pGEM-T and then transformed into competent cells of *Escherichia coli* DH5α, as described by the pGEM®-T Vector Systems (Promega, Madison, WI, USA). The positive clones were sequenced by Beijing Sunbiotech Co., Ltd. (Beijing, China).

## Results

### Identification of non-coding ‘hypothetical ORFs’

First, 1,071, 558, 214, and 59 ‘hypothetical ORFs’ in the circular chromosome, linear chromosome, pAtC58, and pTiC58 plasmids were re-identified using the Z-curve method [19] [20]. With the exception of the putative and ‘hypothetical genes’ in the annotation file, 2,987 function confirmed genes in the four replicons were used to determine the discrimination parameters. The 2,987 genes were randomly divided into two almost equal parts. The first part served as a training set for the calculation of the Fisher coefficients, whereas the other served as a test set for the assessment of algorithm accuracy. Both the training and test sets should include positive and negative samples. In the genome of *A. tumefaciens* C58, 88.6% of the whole DNA sequences are coding sequences, making the preparation of an appropriate set of negative samples quite difficult. Therefore, each of the 2,987 protein-coding genes was randomly shuffled 100,000 times, so that it was transformed into a random sequence. The shuffled sequences then served as negative samples. Sensitivity S_n_ and specificity S_p_ were used to evaluate the algorithm and were defined as follows: S_n_ = TP/(TP+FN), S_p_ = TN/(TN+FP), where TP, TN, FP, and FN are fractions of positive correct, negative correct, false-positive, and false-negative predictions, respectively. Accuracy was defined as the average of S_n_ and S_p_. After performing ten-fold cross-validation tests, the mean sensitivity, specificity, and standard deviation were listed in Table 1. The ‘hypothetical genes’ were re-identified using the final Fisher coefficients and criterion for deciding coding/non-coding. Considering that the negative samples differ in each discrimination process, the recognized non-coding ORFs also have slight differences. The process was repeated 100 times, and the commonly identified non-coding ORFs were adopted. Consequently, 29 ‘hypothetical genes’ in the four replicons of *A. tumefaciens* C58 were identified as non-coding ORFs, which were listed in Table 2.

**Table 1:** Sensitivity, specificity, and accuracy of more than 10-fold cross-validation tests for *A. tumefaciens* C58.

Species	Sensitivity^a^ (%)	Specificity^a^ (%)	Accuracy^b^ (%)
*A. tumefaciens* C58	99.70±0.003	99.97±0.001	99.84

a “±” means standard deviation.

b Accuracy is defined as the average of sensitivity and specificity.

**Table 2:** PIDs of the 29 recognized non-coding ORFs in the replicons of *A. tumefaciens* C58 genome.

Chromosome (or plasmid)			PID			
Chromosome 1	159184472	159184562	159184611	159184672	159184973	15888072
	159184818	159184826	17935555	159184865	159184886	159185410
	159185219					
Chromosome 2	15891663	159185690	159186371	159185840	17937354	159185920
	15890806					
pAtC58	17938747	159186672	159186521	159186542	16119489	159186570
	17939131	17939068	17939097			

### Theoretical and experimental evidence of the recognized ORFs as non-coding ORFs

**Figure 1:**
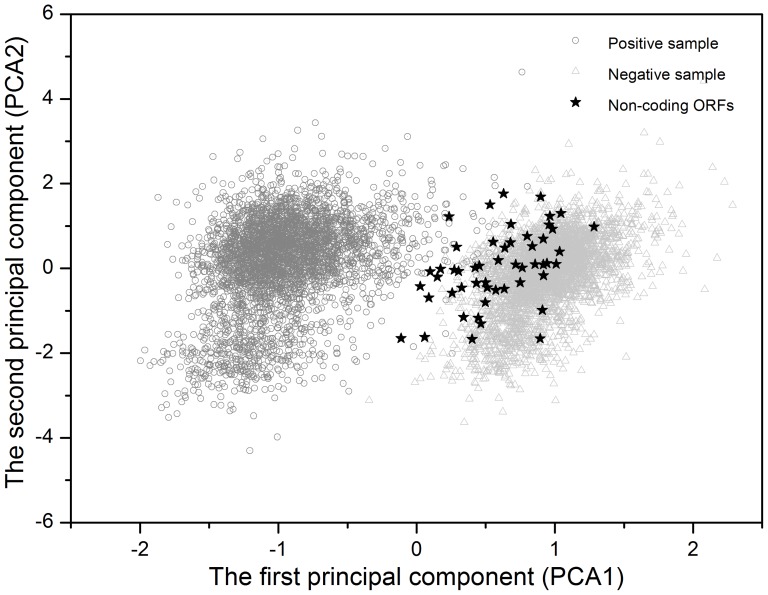
Distribution of points on the principal plane spanned by the first (x) and second (y) principal axes using PCA in *A. tumefaciens* C58. The open circles and triangles represent the function-known genes and corresponding negative samples, respectively. Filled stars represent the recognized non-coding ORFs. The first and second principal axes accounted for 33.4% and 14.9% in *A. tumefaciens* C58 genome of the total inertia of the 21-dimensional space, respectively. Note that the distribution of the open circles is separate from that of the open triangles, suggesting that coding and non-coding sequences are well distinguished. Furthermore, most of the filled stars are far from the core of open circles, implying that the recognized non-coding ORFs listed in Table 2 are unlikely to encode proteins.

In the current annotation of bacterial genomes, false-positive predicted genes always exist, i.e., some randomly occurring ORFs are recognized as protein-coding genes, most of which are relatively short ‘hypothetical ORFs’ [6]–[11]. The difference between protein coding genes and identified non-coding ORFs can be viewed intuitively using principal components analysis (PCA) [23]. Figure 1 shows the distribution of points spanned by the first two principal components on the principal plane for *A. tumefaciens* C58. The coding and non-coding sequences were represented by open circles and triangles, respectively. The first and second principal axes possessed 33.4% and 14.9% of the total inertia of the 21-dimensional space. The first two principal axes were responsible for separating the coding and non-coding sequences into two scarcely overlapping clusters. The recognized non-coding ORFs were represented by filled stars distributed far from the core of function-known genes and close to the random sequences. This implies that the ORFs listed in Table 2 were highly unlikely to encode proteins.

The clusters of orthologous groups of proteins (COG) functional category was added to most of the archaeal and bacterial curated genomic annotations. Each COG is a group of three or more proteins that are inferred to be orthologs. Computational analysis showed that approximately 70% prokaryotic proteins were generally highly conserved and contained ancient conserved regions shared by homologs from distantly related species [24]. Therefore, an ORF that has a COG code is highly likely to be a protein-coding gene. Table 3 showed that approximately 96.8% of function-known genes have COG code. However, for the recognized non-coding ORFs, only 3.8% were assigned with COG codes. In addition, the average lengths of the recognized non-coding ORFs (105.8 aa) were much shorter than that of the function-known genes (357.8 aa). All the above theoretical evidence supports the view that the recognized non-coding ORFs in *A. tumefaciens* C58 were very unlikely to encode proteins.

**Table 3:** Percentage of ORFs with COG code and average length of function-known genes and recognized non-coding ORFs in *A. tumefaciens* C58.

Feature	Genes with known functions	Recognized non-coding ORFs
COG code (%)	96.80	3.85
Average length (aa)	357.78	105.83

**Figure 2:**
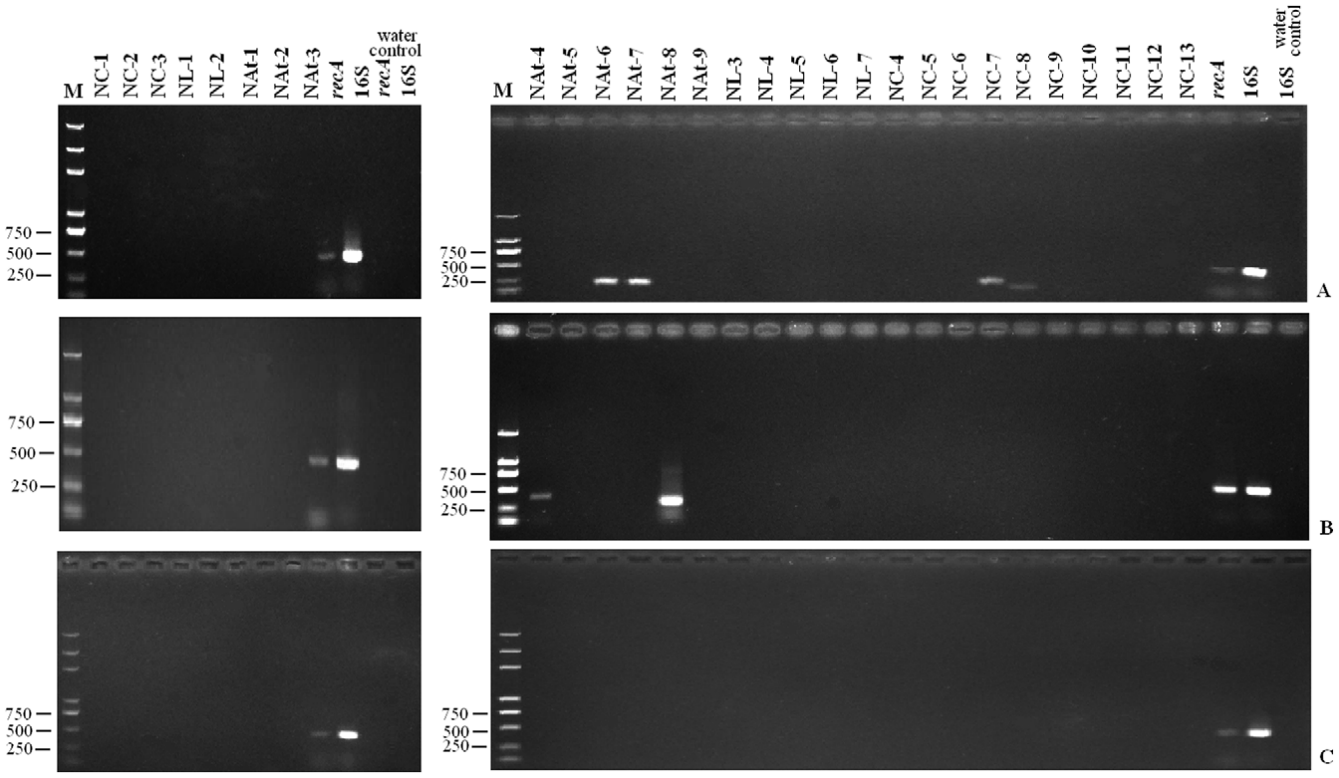
The RT-PCR results of the re-annotated no-coding ORFs. A, B and C represent the RT-PCR results with the cDNA of early log, late log, and stationary stage cells, respectively. For all the three growth stages, the positive controls of *16S rRNA* gene (for multi-copy gene) and *recA* gene (for single-copy gene) were both obtained. (**A**) With the cDNA of the early log stage, NC-7 (210 bp), NC-8 (109 bp), NAt-6 (202 bp), and NAt-7 (194 bp) were successfully amplified. (**B**) With the cDNAs of late log stage, only NAt-4 (374 bp) and NAt-8 (262 bp) were successfully amplified. (**C**) With cDNAs of the stationary stage, all the tested ORFs and the samples without cDNAs (water controls) for *16S rRNA* and *recA* genes, were negative.

To test our theoretical prediction, all the identified 29 non-coding ORFs were verified experimentally. Information of the 29 non-coding ORFs and the designed primers are listed in Table 4, and the RT-PCR results are shown in Figure 2. The PCR using total DNA as each template confirmed that the reagents and primers both work well, and all the ORFs could be amplified precisely (Figure S1). Water was used as a template to detect whether the reagents were contaminated, which yielded negative results (Figure S1). The RT-PCR products from the total RNA sample showed no DNA contamination (Figure S2). After the DNA reagents and the RNA were both confirmed, the cDNA reverse transcribed from the RNA was used as template for PCR. In the RT-PCR results of cDNAs as templates, 16S rRNA and *recA* gene were used as positive control for multi-copy and single-copy genes, respectively (Figure 2). For the early log stage, NC-7 (210 bp), NC-8 (109 bp), NAt-6 (202 bp), and NAt-7 (194 bp) were successfully amplified, whereas NAt-4 (374 bp) and NAt-8 (262 bp) were amplified for the late log stage (Figures 2A and B). For the stationary stage, all the non-coding ORFs showed negative amplification products (Figure 2C). A total of 23 (79%) cDNAs of the tested 29 non-coding ORFs were not amplified, confirming that most predicted non-coding ORFs were not expressed in the three important stages of bacterial growth. DNA sequencing results confirmed that the PCR products were the correct target gene sequences (data not shown). The RT-PCR results verified that the theoretical prediction of non-coding ORFs was very reliable.

**Table 4:** Non-coding sequences and corresponding primers.

Primer Name	PID	Annealing temperature (°C)	Primer sequences
16S rRNA-F	-	50	5′ CGGTAGTCGGAGAAGAAGC 3′
16S rRNA-R	-		5′ CCCAGGCGGAATGTTTA 3′
recA-F	159184959	57	5′ CGGAAGCCCAGAAGAAGG 3′
recA-R			5′ GCGGACGGACGCATAGA 3′
NC-1-F	17935555	57	5′ GCCCTTGCGAGATACGG 3′
NC-1-R			5′ GACGGTGGGTGGGACTTT 3′
NC-2-F	159184886	50	5′ CAGTCCTCGCAGATTTCGC 3′
NC-2-R			5′ CGGCTATTTGCCTTTCG 3′
NC-3-F	159185410	57	5′ TATTGGTCCACATCGTCCTGC 3′
NC-3-R			5′ AAACGGCTGACGGGTGC 3′
NC-4-F	159185219	49	5′ AAGGCCACTCCCTGTCTG 3′
NC-4-R			5′ AGCATCCGCTGTCCAAA 3′
NC-5-F	159184973	50	5′ CGCCGGATGGACCACT 3′
NC-5-R			5′ CGTACCACGCCCACAAAA 3′
NC-6-F	159184865	49	5′ AGTGCCGAAGAGTTGCC 3′
NC-6-R			5′ AGAGGGTTCTGCGATGC 3′
NC-7-F	159184826	50	5′ ATGCCATCGGTCAAATCAA 3′
NC-7-R			5′ CTGAAAGGGCTGTGGAAAA 3′
NC-8-F	159184818	45	5′ GCCTCTTTGCTGCTTTGG 3′
NC-8-R			5′ATCCGACATATTCGTTCTTCACT 3′
NC-9-F	159184672	50	5′ CCGACAGTCACGCAGTTC 3′
NC-9-R			5′ TTGACGACAGTGGATACCG 3′
NC-10-F	159184611	52	5′ TGCGTTTCGTCCTTCACC 3′
NC-10-R			5′ TCGCCCTTTGCCCTTG 3′
NC-11-F	159184562	55	5′ ACGGAAGCGAGTGGTCATT 3′
NC-11-R			5′ ACAGCGGCGCATTTGG 3′
NC-12-F	159184472	48	5′ ATACGCCGATTTCCTCAG 3′
NC-12-R			5′ GACGCCGCTCTTCTTTG 3′
NC-13-F	15888072	50	5′ TCTTCACTAGCTTCACGCCATCT 3′
NC-13-R			5′CGGTTCTGACACCAGGAAACAT 3′
NL-1-F	159186371	50	5′ CGGCGATGAAGGGTGA 3′
NL-1-R			5′ TGAGCGATAGGATTGCAGAG 3′
NL-2-F	15890806	55	5′ GGTTCTGCGGTGTCTTCC 3′
NL-2-R			5′ GCGAGCCATAGCCTTGA 3′
NL-3-F	159185920	50	5′ ACCACCACCCTTACCACT 3′
NL-3-R			5′ TTCCTGCTGCAATGTCC 3′
NL-4-F	17937354	50	5′ TGGTATCCCAATGGTCAA 3′
NL-4-R			5′ GCCCGACAGGAGTTCA 3′
NL-5-F	159185840	55	5′ CTGGCTGCTGGATACGA 3′
NL-5-R			5′ CCTCTTGCGGTTGACTG 3′
NL-6-F	159185690	49	5′ ACGTCGAAGCTGTTTCTTT 3′
NL-6-R			5′ GGCTGTTCACCCTGGTAG 3′
NL-7-F	15891663	49	5′ CGGCAAACTGGAAACAG 3′
NL-7-R			5′ GCAAATGCGAAACAACC 3′
NAt-1-F	17938747	53	5′ CGTCAGGGTCCATTTCACTC 3′
NAt-1-R			5′ CATTGGTATCGCCCGTTTA 3′
NAt-2-F	159186521	53	5′ ACGGTCCTTTCGGTTTGT 3′
NAt-2-R			5′ TCGATTTCCCTTTCACTCAC 3′
NAt-3-F	159186570	53	5′ TCAGCCGATACGCAACTT 3′
NAt-3-R			5′ GTCATGCCTGGAGACGATT 3′
NAt-4-F	17939131	55	5′ TGTCAACAAGCGGAAGAGC 3′
NAt-4-R			5′ ACAACGAGGGTGAGAAGAAA 3′
NAt-5-F	17939097	55	5′ GACCGACTGGTGGAGCAT 3′
NAt-5-R			5′ TGGAAGCAGTTCAAATACCG 3′
NAt-6-F	17939068	55	5′ GATGGCAGGAGGGAAAT 3′
NAt-6-R			5′ GAAATAAGTACGAGGGACGA 3′
NAt-7-F	16119489	50	5′ GGGGAGTTCGTTCATCCG 3′
NAt-7-R			5′ TGCTCTTCACCTTCACCGTAT 3′
NAt-8-F	159186542	50	5′ CTGTATTGCACGCACCAGG 3′
NAt-8-R			5′ CGTCGGGAGGTTCGGTAT 3′
NAt-9-F	159186672	50	5′ CAATAGGCACCGCCACAG 3′
NAt-9-R			5′ AGTCACCGGGTCCAGCAT 3′

### Theoretical prediction and experimental validation of newly predicted protein-coding genes not annotated in NCBI RefSeq

The *ab initio* gene-finding programs used in the NCBI RefSeq annotation pipeline include Glimmer [25], GeneMark [26], and the recently developed Prodigal [21]. However, the Prodigal result has not been incorporated into the RefSeq annotation. FgenesB is another accurate gene prediction program which has not been used in RefSeq annotation. Therefore, Prodigal and FgenesB were used to find new potential protein-coding genes in *A. tumefaciens* C58 genome. Both Prodigal and FgenesB predicted 19 potential new protein-coding genes. BLAST search was performed to find potential functions for these potential protein-coding genes, 13 of which had high sequence similarities with function-known genes in public databases. However, Table 5 showed that in most cases, the query sequences were only aligned to a partial of the BLAST hits. The other 6 were predicted to be ‘hypothetical genes’ with sequence lengths similar to those of their BLAST hits (Table 5).

**Table 5:** Information of the 19 potential new protein-coding genes and their best hit BLAST homologs.

Chromosome	Name	Start	Stop	Strand	Length (aa)	Identity	Subject length (aa)	E value	Function of the subject sequence
Circular	PC-1	446916	447221	+	102	86%	605	2e-31	Phage terminase GpA
chromosome	PC-2	949996	950403	+	136	91%	161	1e-64	Phage major tail protein
	PC-4	1899166	1899594	−	143	95%	142	1e-55	Hypothetical protein
	PC-5	196118	196747	+	210	98%	431	1e-110	D-amino acid dehydrogenase
	PC-6	423064	423597	−	178	95%	177	6e-94	GcrA cell cycle regulator
	PC-7	804614	804946	−	111	63%	120	2e-32	Lysozyme inhibitor
	PC-8	947976	948311	+	112	88%	111	1e-49	Phage head-tail adaptor
	PC-9	1307348	1307650	−	101	82%	101	2e-39	Alkylphosphonate uptake protein
	PC-10	2070741	2071286	+	182	77%	385	8e-73	Glutathionylspermidine synthase
	PC-11	2072921	2073508	+	196	74%	385	6e-84	Glutathionylspermidine synthase
	PC-12	447298	447621	+	108	58%	108	3e-23	Hypothetical protein
	PC-13	947403	947972	+	190	92%	189	2e-95	Hypothetical protein
	PC-14	950403	950762	+	120	88%	118	8e-50	Hypothetical protein
	PC-15	2404305	2404655	−	117	99%	116	1e-58	Hypothetical protein
	PC-16	2643213	2643503	+	97	98%	96	3e-38	Hypothetical protein
Linear	PL-1	934272	935675	+	468	65%	614	4e-176	DNA methylase N-4/N-6 domain
chromosome	PL-2	1122236	1122937	+	234	96%	351	2e-87	Ornithine cyclodeaminase
	PL-3	1048273	1048680	+	136	47%	282	7e-20	GCN5 N-acetyltransferase
	PL-4	1120248	1120820	+	191	95%	351	5e-100	Ornithine cyclodeaminase

**Figure 3:**
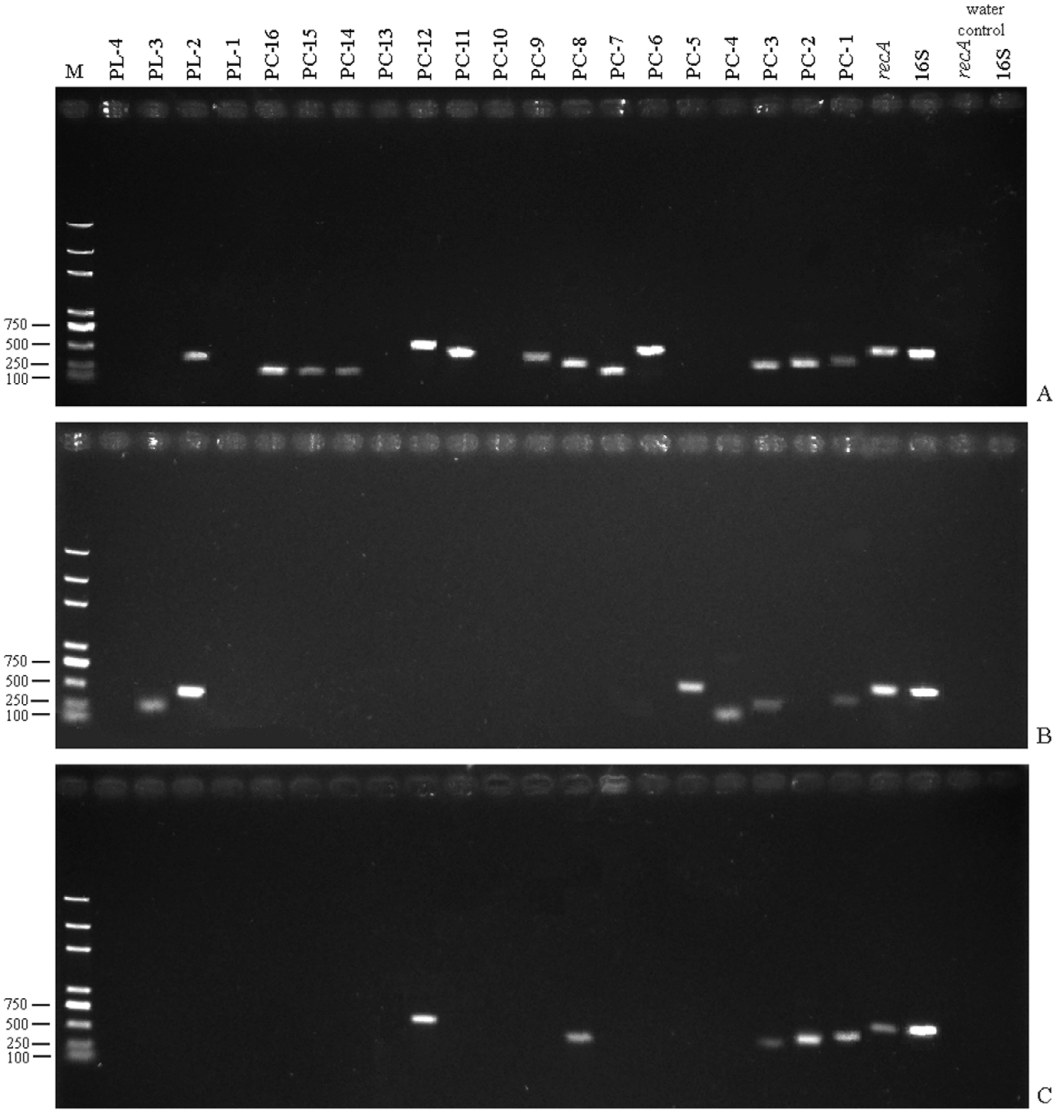
The RT-PCR results of the re-annotated coding ORFs. (**A**) The cDNAs of the early log stage cells, the PC-1 (322 bp), PC-2 (291 bp), PC-6 (341 bp), PC-7 (235 bp), PC-8 (251 bp), PC-9 (325 bp), PC-11 (400 bp), PC-12 (513 bp), PC-14 (238 bp), PC-15 (241 bp), PC-16 (252 bp), and PL-2 (401 bp) were positively amplified. (B) cDNAs of the late log stage, the PC-1 (322 bp), PC-4 (162 bp), PC-5 (456 bp), PL-2 (401 bp), and PL-3 (277 bp) were obtained. (C) The cDNAs of the stationary stage, the PC-1 (322 bp), PC-2 (291 bp), PC-8 (251 bp), and PC-12 (513 bp) were successfully amplified. In all the three growth stages, the positive control of *16S rRNA* gene, *recA* gene, and PC-3 (the positive control gene from *A. tumefaciens* C58) were all obtained. The samples without cDNAs (water controls) for the *16S rRNA* and *recA* genes were all negative.

All of the 19 predicted protein-coding genes underwent experimental verification. The 16S rRNA gene, *recA* gene, and PC-3 gene encoding translation initiation factor IF-2 were selected as positive controls for multi-copy, single-copy, and positive control genes, respectively. Information and the designed primers of the predicted protein-coding genes and three positive controls are listed in Table 6. The RT-PCR results of the cDNA templates are shown in Figure 3, and the control PCR results of the total DNA and total RNA are shown in Figure S3 and Figure S4. All the potential protein-coding genes could be amplified precisely from the total DNA templates (Figure S3), and the RT-PCR products from the total RNA sample had no DNA contamination (Figure S4). In Figure 3, PC-1 (322 bp), PC-2 (291 bp), PC-6 (341 bp), PC-7 (235 bp), PC-8 (251 bp), PC-9 (325 bp), PC-11 (400 bp), PC-12 (513 bp), PC-14 (238 bp), PC-15 (241 bp), PC-16 (252 bp), and PL-2 (401 bp) were positive amplified in the early log stage. PC-1 (322 bp), PC-4 (162 bp), PC-5 (456 bp), PL-2 (402 bp), and PL-3 (276 bp) were amplified in late log stage, whereas PC-1 (322 bp), PC-2 (291 bp), PC-8 (251 bp), and PC-12 (513 bp) were amplified in the stationary stage. Fifteen (79%) genes were successfully amplified in three *A. tumefaciens* C58 growth stages, confirming that they are truly protein-coding genes although they are not annotated in NCBI RefSeq. The DNA sequencing results confirmed that the PCR products were the correct target gene sequences (data not shown). The RT-PCR results verified that the theoretical prediction of novel protein-coding genes was also very reliable.

**Table 6:** The newly predicted protein-coding sequences and the corresponding primers.

Sequence Name	Start	Stop	Annealing temperature (°C)	Primer sequences
16S rRNA-F	-	-	52	5′ CGGTAGTCGGAGAAGAAGC 3′
16S rRNA-R				5′ CCCAGGCGGAATGTTTA 3′
recA-F	-	-	55	5′ CGGAAGCCCAGAAGAAGG 3′
recA-R				5′ GCGGACGGACGCATAGA 3′
PC-3-F^a^	88750	91491	55	5′ TTGCCGCCGCCGAAACT 3′
PC-3-R				5′ TTGCGATCAACGTGACGAAGAAA 3′
PC-2-F	949996	950403	52	5′ ATGGTGGCGCAGAAGGG 3′
PC-2-R				5′ TGCCGAAATCGGGAATGA 3′
PC-1-F	446916	447221	55	5′ CCGCCTGGCTGGTGAAT 3′
PC-1-R				5′ ACCGACAACAACGACGACAA 3′
PC-4-F	1899166	1899584	52	5′ ACGACGGCGATGTGAACC 3′
PC-4-R				5′ TCATTGGCGGTCTTTGCTT 3′
PC-5-F	196118	196747	55	5′ CACCCTCAGCCAGACGACA 3′
PC-5-R				5′ CCCGAAGGCGAACCACA 3′
PC-6-F	423064	423597	50	5′ CAGACGAGCGAGTCGAGAAA 3′
PC-6-R				5′ AACCGCAGAAATGGAAATCA 3′
PC-7-F	804614	804946	50	5′ GCTCAGAGTGGCACTGTTG 3′
PC-7-R				5′ GACGCCGTGTCTTACGC 3′
PC-8-F	947976	948311	49	5′ TCTCGATCCCGGCAAGCT 3′
PC-8-R				5′ GCAACGGCAGACGATGAAGC 3′
PC-9-F	1307348	1307650	55	5′ TCGGGATTGTCGGTCAGG 3′
PC-9-R				5′ AAGCGAGCGGCGAATGG 3′
PC-10-F	2070741	2071286	55	5′ GCACGGCGAACCTTACTG 3′
PC-10-R				5′ CGTCATAGGCGAGATCAAAA 3′
PC-11-F	2072921	2073508	55	5′ GGGCGTTACGCTGACCT 3′
PC-11-R				5′ GCATATCCGAACTCGCTCTT 3′
PC-12-F	447298	447621	55	5′ TGTTCGGCGTAGCGGAGTT 3′
PC-12-R				5′ GGTCACCGGATTGAAGCACC 3′
PC-13-F	947403	947972	50	5′ CGTGATTCTGATTGGCAAGGG 3′
PC-13-R				5′ CGCCGCAGGCTGGTTTT 3′
PC-14-F	950403	950762	55	5′ ATGCCTGAGCGTTTGCGTTAC 3′
PC-14-R				5′ CGATGACCCGGATCATGTCG 3′
PC-15-F	2404305	2404655	52	5′ TCATATTCGGCCTGCACTTC 3′
PC-15-R				5′ CGAGCGGATGGCAAAGG 3′
PC-16-F	2643213	2643503	52	5′ GGTGCTGATCGCCAGTG 3′
PC-16-R				5′ GAAGGAGCGGATGAAGAAG 3′
PL-1-F	934272	935675	51	5′ AGCCGCTACAGAACCTTT 3′
PL-1-R				5′ CGCCTTTGACCGATGT 3′
PL-2-F	1122236	1122937	53	5′ ACAAGCAATATGCGACGATC 3′
PL-2-R				5′ GCAGCAACATGCCGAAC 3′
PL-3-F	1048273	1048680	53	5′ ATCGGTCGAGCGTGGAT 3′
PL-3-R				5′ AGCAGTGCCGTGATGAGAA 3′
PL-4-F	1120248	1120820	50	5′ GCCGAAGTGGTGGAAGAGG 3
PL-4-R				5′ CGCAACGCCGTCAGGAT 3′

a PC-3 gene encodes translation initiation factor IF-2, which is the positive control gene from *A. tumefaciens* C58.

## Discussion

*A. tumefaciens* C58 was the first sequenced genome in *Agrobacterium* species. Therefore, the precise gene annotation for this bacterium is important for microbiological research and plant genetic modification. Considering that most of the ORFs are identified by gene-finding programs, but not verified experimentally in the current stage, many false-positive and several false-negative ORFs always exist in bacterial genome annotation, especially in GC-rich genomes [6]–[15]. Bacterial gene annotation can be considerably improved although it has continuously developed over the past decade. *A. tumefaciens* C58 genome has relatively high GC content, thus it contains fewer overall stop codons and more spurious ORFs.

Many rigorous constraints are imposed on true protein-coding genes rather than randomly occurring ORFs. The generally accepted codon usage pattern is prototype, where ,, and indicate purine, non-guanine, and any bases at the first, second, and third codon positions, respectively [27]–[29]. The first, second, and third codon positions have been suggested to be associated with the biosynthetic pathway, hydrophobicity pattern, and –helix or –strand forming potentiality of the coded amino acids, respectively [27]–[29]. However, the false ORFs do not have such coding constraints. The different codon usage patterns between protein-coding genes and spurious ORFs form the bases of the current algorithm. Figure 1 shows that most of the spurious ORFs were distributed far from the core of the function-known genes, indicating that they do not use a general codon usage pattern. Most of the recognized non-coding ORFs were confirmed to have no transcripts in the three important bacterial growth stages. Although the RT-PCR experimental results under the three tested conditions cannot ensure that these ORFs never express under any conditions, the theoretical evidence obtained from the PCA analysis, COG occupation, and average length distribution provides more compelling evidence. Therefore, these ORFs are highly unlikely to be protein-coding genes. In addition, 15 (79%) of the 19 newly predicted protein-coding genes, were confirmed to be protein-coding genes by RT-PCR. Although the most important problem in bacterial genome annotation is false-positive prediction, the experimental result confirmed that missing genes still exist in *A. tumefaciens* C58. We also noticed that most of the ‘novel genes’ are only aligned to a fraction of the function-known genes in the public databases although the sequence identities were high (Table 5). Detailed functions of these genes should be further investigated. The improved annotation of *A. tumefaciens* C58 will provide more accurate information for the research of this important plant pathogen genome. The re-annotation of *A. tumefaciens* C58 genome can be downloaded from http://211.69.128.148/Atum/. Nucleotide sequence data of the 15 RT-PCR confirmed new genes are available in the Third Party Annotation Section of the DDBJ/EMBL/GenBank databases under the accession numbers TPA: BK008582–BK008596.

## Supporting Information

Methods S1 Identification of non-coding ORFs from annotated hypothetical genes.
(DOC)

Figure S1 The PCR results of 29 DNA fragments re-annotated as no-coding ORFs. The expected products of PCR used total DNA as template were all obtained with the right sizes, 16S rRNA gene (404 bp), *recA* (425 bp), NC-1 (362 bp), NC-2 (437 bp), NC-3 (468 bp), NC-4 (106 bp), NC-5 (127 bp), NC-6 (115 bp), NC-7 (210 bp), NC-8 (109 bp), NC-9 (291 bp), NC-10 (254 bp), NC-11 (111 bp), NC-12 (299 bp), NC-13 (331 bp), NL-1(401 bp), NL-2 (335 bp), NL-3 (242 bp), NL-4 (146 bp), NL-5 (372 bp), NL-6 (124 bp), NL-7 (130 bp), NAt-1 (409 bp), NAt-2 (466 bp), NAt-3 (385 bp), NAt-4 (374 bp), NAt-5 (145 bp), NAt-6 (202 bp), NAt-7 (194 bp), NAt-8 (262 bp) and NAt-9 (128 bp).
(TIF)

Figure S2 The PCR results with total RNA of 29 DNA fragments re-annotated as no-coding ORFs. (**A**) The PCR with RNA of early log phase as templates. (**B**) The PCR with RNA of late log phase as templates. (**C**) The PCR with RNA of stationary phase as templates. When the total RNAs were used as templates in the PCR, no amplification band was produced.
(TIF)

Figure S3 The PCR results of 19 DNA fragments re-annotated as potential protein-coding genes. The expected products of PCR used total DNA of late log phase as templates were all obtained with the right sizes, 16S rDNA (404 bp), *recA* (425 bp), PC-1 (322 bp), PC-2 (291 bp), PC-3 (268 bp), PC-4 (162 bp), PC-5 (456 bp), PC-6 (341 bp), PC-7 (235 bp), PC-8 (251 bp), PC-9 (325 bp), PC-10 (244 bp), PC-11 (400 bp), PC-12 (513 bp), PC-13 (309 bp), PC-14 (238 bp), PC-15 (241 bp), PC-16 (252 bp), PL-1 (376 bp), PL-2 (401 bp), PL-3 (277 bp) and PL-4 (256 bp).
(TIF)

Figure S4 The PCR results with RNA of 19 DNA fragments re-annotated as potential protein-coding genes. (**A**) The PCR with RNA of early log phase as templates. (**B**) The PCR with RNA of late log phase as templates. (**C**) The PCR with RNA of stationary phase as templates. When the total RNAs were used as templates in the PCR, no amplification band was produced.
(TIF)
