# Supplementary material for: Theoretical Prediction and Experimental Verification of Protein-Coding Genes in Plant Pathogen Genome Agrobacterium tumefaciens Strain C58
Source: PLoS One. 2012 Sep 11;7(9):e43176. doi: 10.1371/journal.pone.0043176 (PMC3439454; doi:10.1371/journal.pone.0043176)
Supplement: Methods S1 — Identification of non-coding ORFs from annotated hypothetical genes. (DOC) [file pone.0043176.s001.doc]

**Supplementary Methods S1**

**Identification of non-coding ORFs from annotated hypothetical genes**

The algorithm was briefly described as follows. Frequencies of bases A, C, G and T occurring in an ORF or a fragment of DNA sequence with bases at positions 1, 4, 7, ...; 2, 5, 8, ...; 3, 6, 9, ..., were denoted by *a*_1_, *c*_1_, *g*_1_, *t*_1_; *a*_2_, *c*_2_, *g*_2_, *t*_2_; *a*_3_, *c*_3_, *g*_3_, *t*_3_, respectively. Based on the Z curve, *a*_i_, *c*_i_, *g*_i_, *t*_i_ were mapped onto a point *P*_i_ in a three-dimensional space V_i_, i = 1, 2, 3. The coordinates of *P*_i_, denoted by *x*_i_, *y*_i_, *z*_i_, were determined by the Z-transform of DNA sequence [15].

(1)

The Z-transform of DNA sequence transformed the four frequencies of DNA bases into the coordinates of a point in a three-dimensional space. In addition to the frequencies of codon-position-dependent single nucleotides, the frequencies of phase-specific di-nucleotides were also considered. Let the frequencies of the 16 di-nucleotides *AA*, *AC*, ..., and *TT* of an ORF or a fragment of DNA sequence be denoted by *p(AA)*, *p(AC)*, ..., *p(TT)* respectively. Using the Z-transform

(2)

where *x^X^*, *y^X^* and *z^X^* were the coordinates, *X* = *A*, *C*, *G*, *T*. Let the three-dimensional space V^X^ be spanned by *x^X^*, *y^X^* and *z^X^*. The direct-sum of the subspaces V_1_, V_2_, V_3_, V^A^, V^C^, V^G^ and V^T^ was denoted by a 21-dimensional space V, *i.e.*, V = V_1_$\oplus$V_2_$\oplus$V_3_ $\oplus$V^A^$\oplus$V^C^$\oplus$V^G^$\oplus$V^T^, where the symbol $\oplus$ denoted the direct-sum of two subspaces. The 21 components of the space V, *i.e.*, *u_1_*, *u_2_*, ..., *u_21_*, were defined as follows

(3)

Therefore, an ORF or a fragment of DNA sequence can be represented by a point or a vector in the 21-dimensional space V. Note that *u_i_*[–1, +1], *i* = 1, 2, ..., 21. The space V was a 21-dimensional super-cube with the side length of 2.

To complete the algorithm, two groups of samples are needed, one is a set of positive samples corresponding to protein-coding genes, and the other is a set of negative samples corresponding to non-coding sequences. The two groups of samples constitute the training set used in the Fisher discrimination algorithm. Since non-coding sequences only account for a small fraction of bacterial genomes, it is rather difficult to prepare an appropriate set of non-coding sequences. Therefore, the protein-coding genes were shuffled into random sequences and then served as negative samples. The Fisher linear equation for discriminating the positive and negative samples in the 21-dimensional space V represented a super-plane, described by a vector **c** which had 21 components *c*_1_, *c*_2_, ..., and *c*_21_. Based on the data in the training set, an appropriate threshold *c*_0_ was determined to make the coding/non-coding decision. The threshold *c*_0_ was uniquely determined by making the false negative rate and the false positive rate equal. Once the vector **c** and the threshold *c_0_* were obtained, the decision of coding/non-coding for each ORF was simply made by the criterion of cu > *c_0_* / cu < *c_0_*, where c = (*c*_1_, *c*_2_, ..., *c*_21_)^T^, u = (*u*_1_, *u*_2_, ..., *u*_21_)^T^, and ‘T’ indicated the transpose of a matrix. The criterion of cu > *c_0_* / cu < *c_0_* for making the decision of coding/non-coding can be rewritten as Z(u) > 0/ Z(u) < 0, where Z(u) = cu – *c_0_*. Z(u) was called the Z score or Z index for an ORF or a fragment of DNA sequence.
