# Supplementary figures and images for: Theoretical Prediction and Experimental Verification of Protein-Coding Genes in Plant Pathogen Genome Agrobacterium tumefaciens Strain C58
Source: PLoS One. 2012 Sep 11;7(9):e43176. doi: 10.1371/journal.pone.0043176 (PMC3439454; doi:10.1371/journal.pone.0043176)

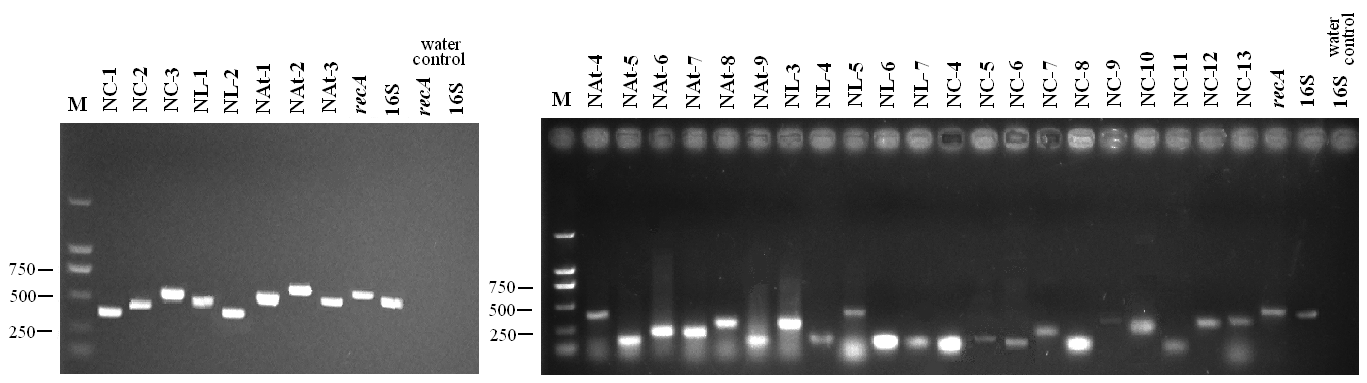

Supplement: Figure S1 — The PCR results of 29 DNA fragments re-annotated as no-coding ORFs. The expected products of PCR used total DNA as template were all obtained with the right sizes, 16S rRNA gene (404 bp), recA (425 bp), NC-1 (362 bp), NC-2 (437 bp), NC-3 (468 bp), NC-4 (106 bp), NC-5 (127 bp), NC-6 (115 bp), NC-7 (210 bp), NC-8 (109 bp), NC-9 (291 bp), NC-10 (254 bp), NC-11 (111 bp), NC-12 (299 bp), NC-13 (331 bp), NL-1(401 bp), NL-2 (335 bp), NL-3 (242 bp), NL-4 (146 bp), NL-5 (372 bp), NL-6 (124 bp), NL-7 (130 bp), NAt-1 (409 bp), NAt-2 (466 bp), NAt-3 (385 bp), NAt-4 (374 bp), NAt-5 (145 bp), NAt-6 (202 bp), NAt-7 (194 bp), NAt-8 (262 bp) and NAt-9 (128 bp). (TIF) [file pone.0043176.s002.tif]

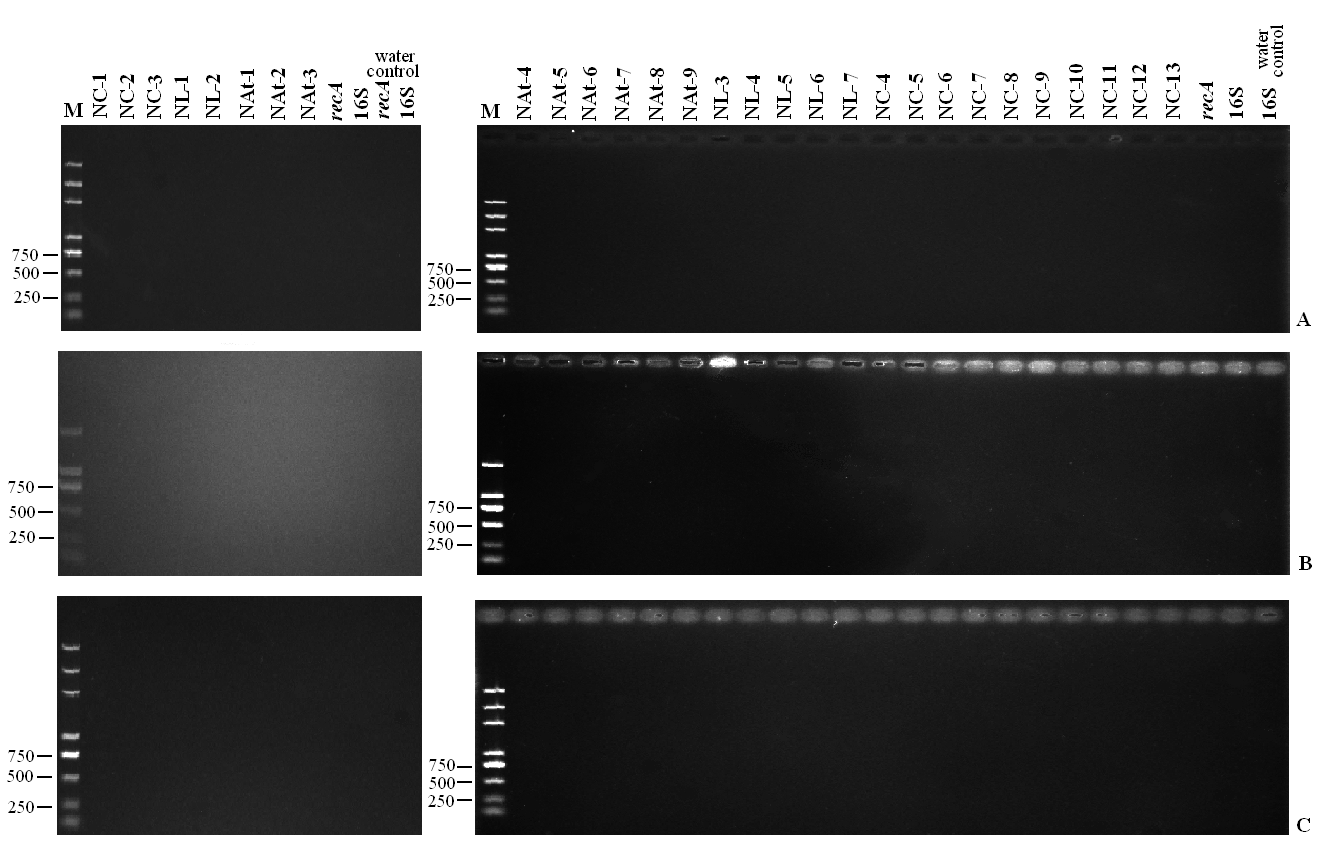

Supplement: Figure S2 — The PCR results with total RNA of 29 DNA fragments re-annotated as no-coding ORFs. (A) The PCR with RNA of early log phase as templates. (B) The PCR with RNA of late log phase as templates. (C) The PCR with RNA of stationary phase as templates. When the total RNAs were used as templates in the PCR, no amplification band was produced. (TIF) [file pone.0043176.s003.tif]

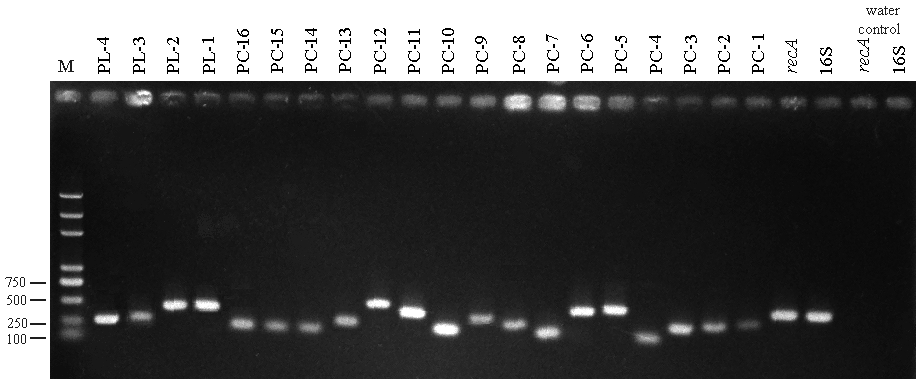

Supplement: Figure S3 — The PCR results of 19 DNA fragments re-annotated as potential protein-coding genes. The expected products of PCR used total DNA of late log phase as templates were all obtained with the right sizes, 16S rDNA (404 bp), recA (425 bp), PC-1 (322 bp), PC-2 (291 bp), PC-3 (268 bp), PC-4 (162 bp), PC-5 (456 bp), PC-6 (341 bp), PC-7 (235 bp), PC-8 (251 bp), PC-9 (325 bp), PC-10 (244 bp), PC-11 (400 bp), PC-12 (513 bp), PC-13 (309 bp), PC-14 (238 bp), PC-15 (241 bp), PC-16 (252 bp), PL-1 (376 bp), PL-2 (401 bp), PL-3 (277 bp) and PL-4 (256 bp). (TIF) [file pone.0043176.s004.tif]

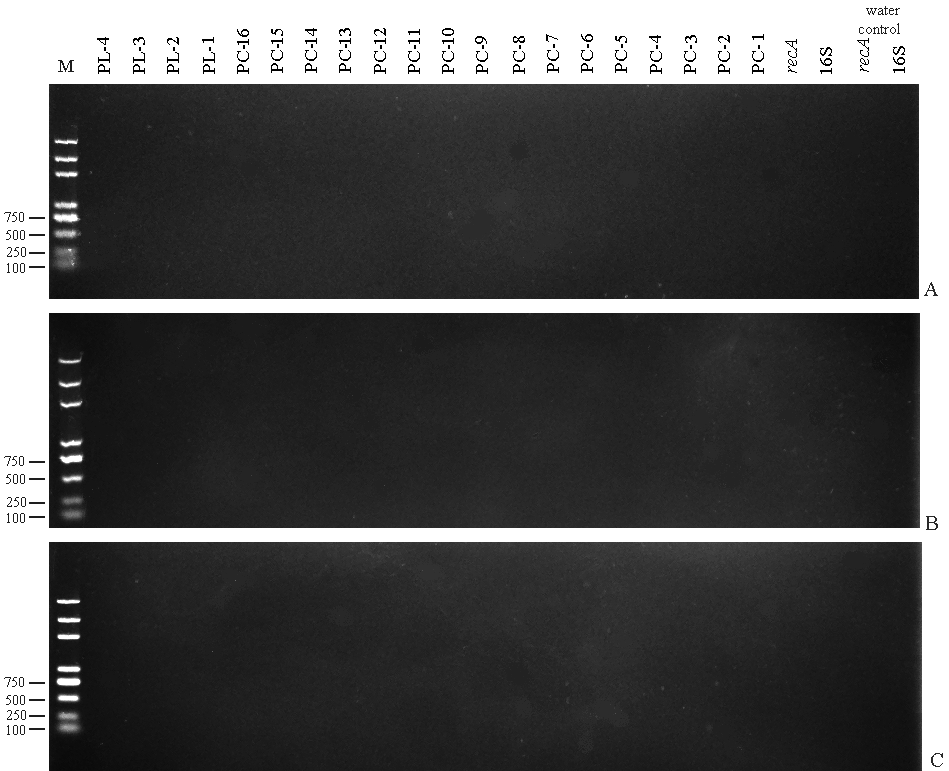

Supplement: Figure S4 — The PCR results with RNA of 19 DNA fragments re-annotated as potential protein-coding genes. (A) The PCR with RNA of early log phase as templates. (B) The PCR with RNA of late log phase as templates. (C) The PCR with RNA of stationary phase as templates. When the total RNAs were used as templates in the PCR, no amplification band was produced. (TIF) [file pone.0043176.s005.tif]
